# Overnight urinary melatonin levels in women with and without HIV: An observational cohort study

**DOI:** 10.1002/brb3.3206

**Published:** 2023-08-07

**Authors:** Helen J. Burgess, Kathleen M. Weber, Ralph Morack, Tsion Yohannes, Jiaqian Xing, Xiaonan Xue, Deborah Gustafson, Anjali Sharma, Elizabeth Daubert, Andrea C. Rogando, Audrey L. French

**Affiliations:** ^1^ Department of Psychiatry University of Michigan Ann Arbor Michigan USA; ^2^ Hektoen Institute of Medicine/CORE Center of Cook County Health Chicago Illinois USA; ^3^ Department of Epidemiology and Population Health Albert Einstein College of Medicine Bronx New York USA; ^4^ Department of Neurology State University of New York Downstate Health Sciences University Brooklyn New York USA; ^5^ Department of Medicine Albert Einstein College of Medicine Bronx New York USA; ^6^ College of Science and Health Charles R. Drew University of Medicine and Science Los Angeles California USA; ^7^ Department of Medicine Stroger Hospital of Cook County Health Chicago Illinois USA

**Keywords:** actigraphy, HIV, melatonin, sleep

## Abstract

**Introduction:**

Despite significant improvements in longevity and quality of life associated with antiretroviral therapy, individuals with HIV still suffer from a higher burden of sleep and circadian disruption and inflammatory‐based diseases than individuals without HIV. While melatonin is a hormone that has a role in sleep and circadian regulation and has anti‐inflammatory properties, the overnight concentration of the urinary melatonin metabolite has not yet been reported in people with HIV.

**Methods:**

The aim of this study was to compare the overnight urinary melatonin metabolite levels in women aged 35–70 years with HIV (*n* = 151) to a well‐matched comparison group of women without HIV (*n* = 147). All women wore a wrist actigraphy monitor and completed daily diaries documenting sleep timing and use of medications and drugs or alcohol for 10 days. Participants collected their overnight urine near the end of the monitoring period.

**Results:**

Melatonin levels did not differ between women with or without HIV, but more than 40% of women had low levels of melatonin. Higher body mass index predicted lower levels of melatonin, and lower levels of melatonin were associated with lower sleep efficiency as assessed with wrist actigraphy.

**Conclusion:**

These data lay the foundation for exploration of the longitudinal consequences of endogenous melatonin levels for inflammatory‐based diseases in aging women with and without HIV. Future studies should consider the use of supplemental melatonin to improve sleep in women with lower levels of melatonin.

## Graphical abstract

While melatonin is a hormone that has a role in sleep and circadian regulation and has anti‐inflammatory properties, the overnight concentration of the urinary melatonin metabolite has not yet been reported in people with HIV. The aim of this study was to compare the overnight urinary melatonin metabolite levels in women aged 35–70 years with HIV (*n* = 151) to a well‐matched comparison group of women without HIV (*n* = 147). Melatonin levels did not differ between women with or without HIV, but more than 40% of women had low levels of melatonin.

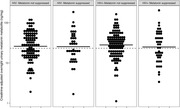

## 1 INTRODUCTION

Despite significant improvements in longevity and quality of life associated with antiretroviral therapy (ART), individuals with human immunodeficiency virus (HIV) still suffer from a higher burden of sleep and circadian disruption and inflammatory‐based diseases than individuals without HIV (Deeks et al., 2013; Jean‐Louis et al., 2012; Lee et al., 2012). Women with HIV and those older than 40 years may be especially affected (Nasi et al., 2017; Wu et al., 2015). The pathophysiology of sleep disturbance in those living with HIV is still largely unknown, but possibilities include an impact of HIV itself on CNS functioning, side effects of antiretroviral medications, mental health issues, and substance abuse (Wu et al., 2015). Melatonin is a hormone that has a role in sleep and circadian regulation and has anti‐inflammatory properties (Cho et al., 2021; Reiter et al., 2017). Melatonin is almost exclusively produced by the pineal gland, and plasma melatonin concentrations are typically low throughout the day, with a rapid rise 1–3 h before habitual bedtime, elevation throughout the nocturnal sleep episode, and a rapid decline approximately 1 h after habitual wake time. Salivary melatonin concentrations are approximately threefold lower than plasma concentrations but show similar timing to plasma melatonin levels (Voultsios et al., 1997). In addition, plasma melatonin is rapidly metabolized, mainly in the liver, and measurement of the principal metabolite of melatonin, 6‐sulfatoxymelatonin, which is excreted in urine, reflects approximately 70% of the total plasma melatonin produced from the previous night (Graham et al., 1998). Large prospective research studies have indicated that lower levels of the urinary melatonin metabolite increase the risk for later developing inflammatory‐based diseases such as prostate cancer (Sigurdardottir et al., 2015), breast cancer (Schernhammer & Hankinson, 2009), and cardiovascular (Forman et al., 2010) and cardiometabolic disease (McMullan et al., 2013).

We identified only three studies to date that have examined melatonin levels in individuals with HIV. In one study, 77 individuals with HIV who had received ART for less than 1 month and 30 controls without HIV gave a single nighttime blood sample between 12:00 a.m. and 3:00 a.m., which was assayed for serum melatonin (Nunnari et al., 2003). They found significantly lower levels of melatonin in the individuals with HIV compared to the controls (Nunnari et al., 2003). In another study, a single saliva sample was collected in the morning between 8:00 a.m. and 10 a.m. from 49 individuals with HIV and 49 controls without HIV (Ahmadi‐Motamayel et al., 2017). In that study, the group with HIV consisted of more women and was older than the controls, and not all were taking antiretroviral medications. The authors reported significantly lower levels of melatonin in the individuals with HIV compared to the controls without HIV, although this group difference was no longer significant when the group comparison was adjusted for age and sex (Ahmadi‐Motamayel et al., 2017). In a third study, 96 people with HIV who were taking antiretroviral medications and considered to have advanced AIDS had a blood sample collected in the morning between 7:00 a.m. and 8:00 a.m. (Wang et al., 2014). The authors found that the cerebrospinal concentration of the HIV Tat protein was positively correlated with the concentration of melatonin in the morning blood sample (Wang et al., 2014). This finding was unexpected as the HIV Tat protein triggers tryptophan catabolism that would be expected to lead to lower levels, rather than higher levels of melatonin in people with HIV (Samikkannu et al., 2009).

All these prior studies attempted to assess nocturnal melatonin secretion through a single saliva or blood sample collection. However, the overall secretion of melatonin at night is most commonly assessed by measuring the concentration of the melatonin metabolite, 6‐sulfatoxymelatonin, in an overnight urine sample. The overnight concentration of the urinary melatonin metabolite has not yet been reported in HIV. Thus, the primary aim of this study was to compare the levels of the overnight urinary melatonin metabolite in a larger sample of people with HIV and a comparison group of demographically similar people without HIV. Based on the mixed findings in the literature, we hypothesized that the concentration of the melatonin metabolite would be similar in individuals with and without HIV infection. Secondary aims of the study were to examine predictors of melatonin concentrations and assess the association between melatonin concentration and subjective and objective markers of sleep.

## 2 MATERIALS AND METHODS

### 2.1 Participant eligibility and recruitment

Women were primarily recruited from the Chicago, Bronx, and Brooklyn sites of the Women's Interagency HIV Study (WIHS), and additionally from a clinical care setting at the Chicago site. The WIHS is a longitudinal observational cohort study of women with HIV and demographically similar women without HIV initiated in 1994 that has been described elsewhere (Adimora et al., 2018; Bacon et al., 2005). Briefly, the WIHS, and subsequently the MACS/WIHS Combined Cohort Study (MWCCS), collected demographic, behavioral, medication, and health history data, conducted physical exams, and performed phlebotomy at least annually.

**FIGURE 1:**
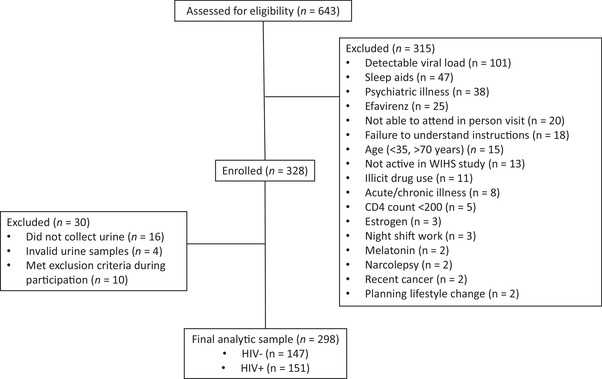
A STROBE study flow chart.

Women were eligible for this study if they were aged 35–70 years and English speaking. Inclusion criteria for women with HIV included stable ART use, an undetectable viral load (defined as HIV RNA level <200 copies/mL), and CD4+ T lymphocyte count ≥200 cells/mm^3^ in the 6 months prior to enrollment. Exclusion criteria for all women included a recent (past 6 months) severe acute or chronic uncontrolled medical condition, psychiatric illness; narcolepsy; current illicit drug use (>1 day/week, assessed via self‐report) other than marijuana (i.e., crack, cocaine, heroin, methamphetamines, hallucinogens); current use of the following medications: efavirenz (disturbs sleep), prescription hypnotics, over‐the‐counter sleep aids >2 nights per week, melatonin supplementation in the past month, estrogen‐containing hormonal contraceptives, or hormonal replacement therapy (all confound melatonin assessment); currently pregnant, lactating, or <3 months postpartum; night shift work (including sex work) >2 nights per week; and travel to a new time zone in the past month. All women were instructed not to use illicit drugs, sleep aids, and supplemental melatonin during the 10‐day study. Enrollees were also instructed not to consume nonsteroidal anti‐inflammatory drugs (NSAIDs) in the 3 days and alcohol in the 24 h before overnight urine collection as these drugs are known to suppress melatonin (Ekman et al., 1993; Surrall et al., 1987). The main reasons for ineligibility among the 315 WIHS participants screened but excluded from the study were detectable HIV viral load at last visit, use of sleep aids, and severe chronic mental illness (Figure 1). Recruitment and study visits occurred from October of 2018 through January 2020. The study was performed according to the principles outlined by the Helsinki Declaration. All participants provided written informed consent prior to their participation, and the study was conducted with approval from the institutional review board at each site (Chicago—County Health IRB 18‐008; Brooklyn—SUNY Downstate Health Sciences University IRB & Privacy Board 1280378‐8; Bronx—Albert Einstein College of Medicine IRB 2018–9115).

### 2.2 Study procedures

For most participants, the 30‐min study visit occurred at the end of a scheduled WIHS research visit. Women completed additional sleep questionnaires, and each participant was asked to continuously wear a wrist actigraphy monitor (30‐s epochs; Actiwatch Spectrum, Respironics) on their nondominant wrist and press the event marker on the monitor before and after sleep each night for 10 days. Participants were also instructed to complete a daily diary, documenting sleep timing and use of medications and drugs or alcohol during the 10 days. Participants received a urine collection kit and were instructed to completely empty their bladder before going to bed and then collect their overnight urine (any urine voided during middle of the night and first thing in the morning) (Schernhammer et al., 2004) during one of the last nights (between days 7 and 9) of wrist actigraphy monitoring. The urine sample was refrigerated at home and during transport to the research clinic in an insulated bag with ice pack. During this return study visit, the activity data and sleep diary were also reviewed by research staff with the participant. Women received compensation for study participation and study‐related travel.

Demographic data were gathered using WIHS survey instruments (Adimora et al., 2018; Bacon et al., 2005). We assessed age, race, ethnicity, education level, marital status, average annual household income, employment status, alcohol use, cigarette use, marijuana use, illicit drug use, depressive symptoms (Center for Epidemiologic Studies Depression Scale [CES‐D]), posttraumatic stress symptoms (PTSD Checklist for DSM‐IV), menopausal status (menstrual phase was not tracked), and body mass index (BMI). For women with HIV, CD4 counts were measured at the study visit. Nadir CD4 count and years on ART were calculated from available research or care records. For the purposes of analysis, HIV viremia was defined as HIV RNA ≥20 copies/mL.

### 2.3 Assessments of melatonin and sleep

#### 2.3.1 Melatonin

The urine samples were refrigerated at the New York sites and shipped on cold packs to the Chicago site for processing, freezing, and analysis. All samples were assayed in duplicate. 6‐Sulfatoxymelatonin (aMT6s) was assayed using the Buhlmann enzyme‐linked immunosorbent assay (ELISA), with a lower limit of detection of 0.8 ng/mL. Intraassay and interassay coefficients of variation for higher levels of the melatonin metabolite (∼30 ng/mL) are 5.3% and 8.4%, respectively. Creatinine levels were assayed using the R&D Systems Creatinine Parameter ELISA, which enabled creatinine standardization to account for differences in urine concentration. The lower limit of detection for this assay is 0.07 mg/dL. Intraassay and interassay coefficients of variation for higher levels of creatinine (∼9 pg/mL) are 3.4% and 4.0%, respectively. Laboratory personnel were blinded to participant serostatus, and samples from all participants were assayed together in batches and quality control samples were included in each batch. A coefficient of variance cutoff of 10% was used to determine replicate precision—if the replicates had a coefficient of variation (CV) below this value, the mean of the replicates was used in analyses.

#### 2.3.2 Sleep

##### Actigraphy‐derived sleep measures

The wrist actigraphy data were analyzed with the Actiware 6.1.2 program (Respironics). The setting of nightly rest intervals for analysis was guided by the event markers, sleep diary, light data, and activity levels (Patel et al., 2015). Objective actigraphic estimates of sleep onset time (clock time of the first epoch scored as sleep in each rest interval), final wake time (clock time of the last epoch scored as sleep in each rest interval), total sleep time (number of minutes scored as sleep in each rest interval), and sleep efficiency (proportion of time between sleep onset and final wake time scored as sleep in each rest interval, expressed as a percentage) were extracted for each study day and then averaged for the first 7 days of data collection. Participants with at least three consecutive nights of actigraphy data were included (Lee et al., 2012).

##### Sleep questionnaires

Three sleep‐related questionnaires were completed at study enrollment: the Pittsburgh Sleep Quality Index (PSQI), a recall of sleep quality in the past month (score >5 indicates poor sleep quality) (Buysse et al., 1989); the Insomnia Severity Index, which quantifies perceived insomnia severity (score of 15–21 indicates clinical insomnia, 22–28 indicates severe clinical insomnia) (Okun et al., 2009); and the Epworth Sleepiness Scale, which measures daytime sleepiness (score >11 indicates “excessive sleepiness”) (Johns, 1991).

### 2.4 Statistical methods

Comparisons between women with and without HIV were evaluated using chi square tests for categorical variables and Wilcoxon rank‐sum test for continuous variables. Due to a skewed distribution, the creatinine‐adjusted urinary melatonin (CAUM) metabolite (6‐sulphatoxymelatonin) values were log transformed (Schernhammer et al., 2004). A multivariate linear regression analysis predicting melatonin on the log scale was used to determine which of these variables were associated with melatonin levels: HIV group, age, BMI, menopausal status (yes/no), race (Black vs. non‐Black), habitual at‐risk alcohol drinker (>7 drinks/week vs. not), current smoker (yes/no), current illicit drug use (yes/no), depression (CES‐D ≥ 16), and use of substances that suppress melatonin (alcohol within 24 h, NSAIDs within 72 h before urine collection, daily use of beta blockers), while adjusting for photoperiod (daylength) on the day of urine collection. Associations between each of the sleep variables and the melatonin metabolite were examined using Spearman rank correlations. All analyses were conducted using R (Version 3.6.3, The R Foundation for Statistical Computing) and significance was based on a two‐sided *p* < .05.

### 2.5 Sample size and statistical power

With 151 HIV+ and 147 HIV– women, the study had 80% power to detect a minimum of 0.3 *SD* difference in melatonin (or on log scale) between HIV+ and HIV– women. Thus, if there was any clinical meaningful difference in melatonin between HIV+ and HIV– women, the study was adequately powered to detect that (Cohen, 1992). The study also had 80% power to detect a minimum slope of 0.16 between melatonin (or on log scale) and any continuous variable in the linear regression model.

## 3 RESULTS

### 3.1 Characteristics of study cohort

Of the 328 women enrolled in the study, 312 (95%) collected their urine, although four women provided invalid samples that were not collected properly and were not included. Of the 308 who collected their urine correctly, 10 women were removed from the analysis due to the use of a nightly sleep aid, a major acute medical illness or engagement in night shift work >2 nights/week during the 10‐day study. Thus, the final sample of melatonin metabolite data was generated from 298 women (Figure 1). Descriptive data for these participants are presented in Table 1. Despite the inclusion criteria of clinical viral suppression (defined as HIV RNA level <200 copies/mL) in the 6 months prior to enrollment, 26 women were identified as being viremic (>20 copies/mL) during the study. Melatonin metabolite levels did not differ significantly between women with HIV who were aviremic versus viremic (*p* = .74), and therefore these two groups were combined to yield one sample of women with HIV infection. Overall, the study participants broadly reflect the demographics of urban women aging with HIV in the United States with a well‐matched comparison group of women without HIV. The mean age of the entire sample was 52.6 years, most women were Black (75%), 40% had less than a high school education, 69% had an annual income less than $18,000, and 36% were employed. The majority of the women were menopausal (67%). Women with and without HIV infection were similar in terms of race, ethnicity, education level, married/cohabiting status, income, employment, use of marijuana, illicit drugs and beta‐blockers, clinically relevant depressive and PTSD symptoms, menopausal status, and BMI. The women without HIV were younger on average by 1.8 years than the women with HIV and reported higher alcohol and cigarette use (Table 1).

**TABLE 1:** Characteristics of study participants by human immunodeficiency virus status.

	HIV– (*N* = 147)	HIV+ (*N* = 151)	*p*
City			.48
Chicago, *N* (%)	79 (53.7)	74 (49.0)	
New York City, *N* (%)	68 (46.3)	77 (51.0)	
Age at visit, years	51.7 ± 7.8	53.5 ± 7.6	.04
Race/ethnicity			.14
Black (Non‐Hispanic), *N* (%)	112 (77.2)	110 (72.8)	
White (Non‐Hispanic), *N* (%)	3 (2.1)	5 (3.3)	
Hispanic, *N* (%)	30 (20.7)	31 (20.5)	
Other (Non‐Hispanic), *N* (%)	0 (0)	5 (3.3)	
Education attained			.91
Less than high school, *N* (%)	55 (41.4)	58 (38.9)	
High school, *N* (%)	41 (30.8)	47 (31.5)	
College and above, *N* (%)	37 (27.8)	44 (29.5)	
Married/cohabiting, yes, *N* (%)	35 (29.4)	35 (24.3)	.43
Annual Income, <$18,000, *N* (%)	80 (67.2)	100 (70.4)	.67
Employed, *N* (%)	55 (41.7)	45 (30.2)	.06
Alcohol use			<.001
Abstainer, *N* (%)	59 (44.7)	92 (61.7)	
>0–7 drinks/week, *N* (%)	53 (40.2)	54 (36.2)	
>7 drinks/week, *N* (%)	20 (15.2)	3 (2.0)	
Cigarette use, current, *N* (%)	66 (50.0)	45 (30.2)	.001
Marijuana use, current, *N* (%)	34 (28.6)	27 (18.8)	.08
Illicit drug use, current, *N* (%)	8 (6.7)	5 (3.5)	.36
Beta‐blocker use, current, *N* (%)	24 (16.3)	29 (19.2)	.62
Depression (CES‐D) ≥16, *N* (%)	16 (13.6)	34 (23.6)	.06
PTSD (PCL‐C) ≥34, *N* (%)	40 (33.9)	36 (26.1)	.22
Menopausal, yes, *N* (%)	81 (61.4)	107 (71.8)	.08
Body mass index, kg/m^2^	31.7 ± 7.2	32 ± 7.7	.87
Women with HIV only			
CD4 T cell count, cells/mm^3^		747.7 ± 342.5	N/A
Nadir CD4 count, cells/mm^3^		225.5 ± 163.3	N/A
Years on ART, median (range)		17 ± 5.7	N/A

*Note*: Data are presented as *N* (%) or mean ± *SD* unless otherwise indicated; *p*‐values are from Chi‐squared test or Wilcoxon rank‐sum test, where appropriate.

Abbreviations: ART, antiretroviral therapy; CES‐D, Center for Epidemiologic Studies Depression Scale; HIV, human immunodeficiency virus; PCL‐C, PTSD Checklist for DSM‐IV.

### 3.2 Melatonin metabolite

**FIGURE 2:**
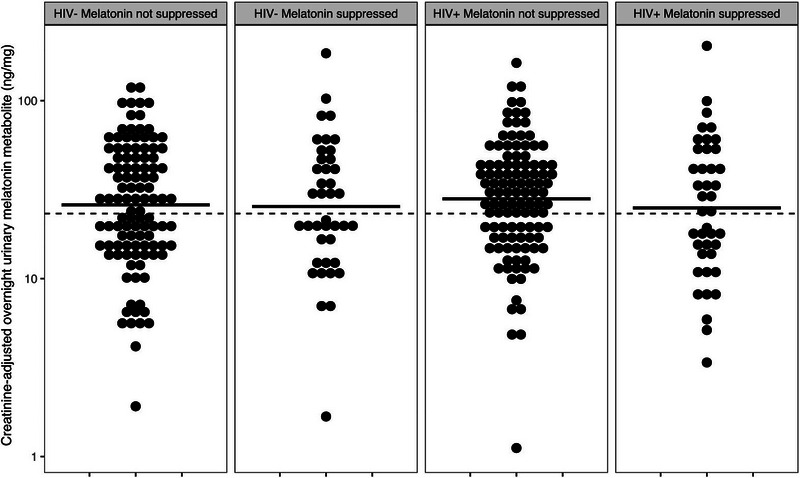
Dot plots of creatinine‐adjusted overnight urinary melatonin levels in women with and without HIV. Women who reported using substances that suppress melatonin (use of beta‐blockers, alcohol in 24 h, and/or NSAID use in 72 h before urine collection) are shown separately in each serostatus group.

The levels of creatinine‐adjusted melatonin metabolite (6‐sulphatoxymelatonin) in the entire sample were similar between the two HIV groups (Figure 2). Importantly, 53 women were taking daily beta‐blockers, reflecting common use in this population. Nine of these women and 28 additional women reported taking an NSAID 3 days before urine collection and/or alcohol 24 h before urine collection on their daily diaries despite instructions (Figure 2). The multivariate‐adjusted regression analysis revealed that melatonin levels did not differ by HIV serostatus (Table 2). Also, while melatonin levels were lower in women who reported use of substances that suppress melatonin (alcohol within 24 h, NSAIDs within 72 h before urine collection, daily use of beta blockers), these differences were not statistically significant (Figure 2; Table 2). Indeed, in the regression analysis, BMI was the only significant predictor of melatonin, where higher BMI was associated with lower levels of melatonin (Table 2). Of note, 40% of the women with HIV and 47% of the women without HIV had levels of urinary melatonin that were less than 23.3 ng/mg, which is the reported lower 95% confidence interval (CI) of urinary melatonin levels in healthy women aged 50–60 years old (Braam & Spruyt, 2022).

**TABLE 2:** Tested predictors of creatinine‐adjusted melatonin metabolite levels.

	Log creatinine‐adjusted melatonin metabolite (*n* = 298)	
	Adjusted beta (95% CI)	*p*
HIV serostatus (with HIV)	0.02 (−0.19, 0.22)	.87
Age (years)	−0.004 (−0.02, 0.01)	.65
Body mass index (kg/m^2^)	−0.02 (−0.03, −0.003)	.018
Menopausal status (yes)	−0.17 (−0.44, 0.10)	.22
Race (Black)	−0.003 (−0.24, 0.24)	.98
Alcohol at risk drinker (> 7 drinks/week)	−0.17 (−0.53, 0.19)	.36
Current smoker (yes)	−0.17 (−0.37, 0.04)	.12
Current drug user (yes)	−0.36 (−0.87, 0.14)	.16
Depression (CES‐D) (≥16)	−0.08 (−0.33, 0.18)	.56
Use of substances suppress melatonin (yes)	−0.02 (−0.24, 0.20)	.86

*Note*: The model was adjusted for photoperiod (daylength).

### 3.3 Objective and subjective sleep variables

Of the 298 women in the final sample, 281 also wore the wrist monitor for at least 3 consecutive nights. Of those with at least 3 consecutive nights of data collected, 21 women collected 3–6 nights of actigraphy data (7.5%), with the remainder collecting 7 consecutive nights of data (92.5%). The sleep variables are shown in Table 3. The only significant difference between the HIV groups was slightly later sleep timing, by about 30 min, in the group without HIV.

**TABLE 3:** Objective and subjective sleep variables by human immunodeficiency virus status.

	HIV– (*N* = 147)	HIV+ (*N* = 151)	*p*
Wrist actigraphy measures			
Sleep onset time (dec. time)	24.1 ± 1.8	23.5 ± 1.6	0.02
Final wake time (dec. time)	7.5 ± 1.6	7.1 ± 1.4	0.01
Total sleep time (h)	6.4 ± 1.3	6.4 ± 1.1	0.54
Sleep efficiency (%)	85.8 ± 6.1	85.7 ± 6.7	0.87
Sleep questionnaires			
Pittsburgh Sleep Quality Index	6.3 ± 3.8	6.6 ± 3.8	0.56
Insomnia Severity Index	6.3 ± 5.8	7.1 ± 5.9	0.18
Epworth Sleepiness Scale	7.4 ± 4.7	7.5 ± 4.7	0.88

*Note*: Data are presented as mean ± *SD*; *p*‐values are from Wilcoxon rank‐sum test.

### 3.4 Associations between melatonin metabolite and sleep variables

Correlations between the CAUM metabolite and the sleep variables are shown in Table 4. In both women with and without HIV, lower levels of the melatonin metabolite were modestly associated with poorer sleep efficiency (worse sleep continuity) as measured with wrist actigraphy. Only in the women with HIV were lower levels of the melatonin metabolite associated with earlier sleep times and more daytime sleepiness.

**TABLE 4:** Correlations between creatinine‐adjusted melatonin metabolite and sleep variables by human immunodeficiency virus status.

	HIV– (*N* = 147)	HIV+ (*N* = 151)
Creatinine‐adjusted melatonin metabolite		
Wrist actigraphy measures		
Sleep onset time (dec. time)	−0.02	0.19^*^
Final wake time (dec. time)	−0.04	0.17^*^
Total sleep time (h)	0.03	0.08
Sleep efficiency (%)	0.21^*^	0.28^*^
Sleep questionnaires		
Pittsburgh Sleep Quality Index	−0.04	−0.16
Insomnia Severity Index	−0.01	−0.16
Epworth Sleepiness Scale	−0.03	−0.22^*^

*Note*: The unadjusted Spearman correlations at *p* < .05 are bolded and asterisked.

## 4 DISCUSSION

To our knowledge, this is the first study to compare overnight urinary melatonin levels in women with and without HIV. In this large, well‐matched study of women with and without HIV, we found that urinary melatonin levels did not significantly differ by HIV serostatus. These results are consistent with the lack of an HIV serostatus group difference in melatonin levels as reported by Ahmadi‐Motamayel and colleagues after they adjusted their group comparison for age and sex differences (Ahmadi‐Motamayel et al., 2017). However, the results are not consistent with those of Nunnari and colleagues who found *lower* levels of melatonin in individuals with HIV versus those without HIV (Nunnari et al., 2003), nor with Wang and colleagues (Wang et al., 2014) whose results suggested that higher HIV viral activity (HIV Tat protein) would be associated with *higher* melatonin levels (Samikkannu et al., 2009). Notably the women with HIV in our sample had been taking ART for many years, with the majority demonstrating excellent HIV viral control, whereas in these earlier studies the participants were either not taking ART or had only started recently and thus likely had higher HIV viral loads. Even in the small number of viremic women in our sample, the viral load was very low (median: <66 copies/mL). Thus, our results suggest that in women with well‐controlled HIV infection, melatonin levels do not differ from those observed in similar women without HIV.

Overall, the women with and without HIV were well matched on sociodemographic factors. Race/ethnicity, education, marital/cohabiting status, income, employment, menopausal status, BMI, and use of marijuana, illicit drugs, and beta‐blockers were not significantly different between the two groups. Psychosocial variables such as depression and PTSD symptoms were also similar between the two groups. Although the women with HIV were slightly older, they used significantly less alcohol and cigarettes than the women without HIV. This group difference may result from more medical and support services, and increased self‐care following a diagnosis of HIV, but could also be due to a survival bias in the women with HIV (Chandran et al., 2019). While the higher use of alcohol and cigarettes in the women without HIV could have suppressed melatonin levels and reduced the difference in melatonin levels between the serostatus groups, this appears unlikely as these factors were not found to predict melatonin levels (Table 2). Indeed, even the daily use of substances that suppress melatonin (daily use of beta blockers [Stoschitzky et al., 1999], alcohol within 24 h [Ekman et al., 1993], NSAIDs within 72 h before urine collection [Surrall et al., 1987]) as coded from the daily diaries was associated with lower levels of melatonin, but this was not statistically significant (Figure 2; Table 2). Likewise, the effects of menopausal status and race on melatonin levels (Burgess & Fogg, 2008; Jeong et al., 2019) were in the expected direction, but were not statically significant. Only BMI significantly predicted urinary melatonin levels, with higher BMI associated with lower levels of melatonin as reported previously (Burgess & Fogg, 2008).

Notably, regardless of HIV serostatus, about 40%–50% of the women in this study secreted low levels of melatonin (<23.3 ng/mg, the lower end of 95% CIs reported in healthy 50–60 years old women [Braam & Spruyt, 2022]). In addition, we found in both serostatus groups that lower levels of urinary melatonin were associated with lower levels of objectively measured sleep efficiency, reflecting worse sleep continuity and more sleep disturbance (Table 4). In the presence of lower levels of melatonin, some studies have reported that supplemental melatonin or melatonin agonists can improve sleep continuity (Garfinkel et al., 1995; Scheer et al., 2012). Thus, future studies should consider the use of supplemental melatonin to improve sleep in those with lower levels of melatonin.

Limitations of this study include that the sample only consisted of women and may not reflect the relationship between endogenous melatonin levels and HIV serostatus in men. In addition, the middle‐aged women in our sample were living in socioeconomically distressed communities, and thus results may not reflect melatonin levels in younger or older women or in those living in more favorable socioeconomic circumstances. Three quarters were Black, more than one third had less than a high school education, and only one third were employed. We also intentionally sought to study women with well‐controlled HIV as that is the clinical treatment goal. However, these women reflect the demographics of women aging with HIV in the United States. Lastly, our inclusion/exclusion criteria led us to enroll medically stable women who were not taking sleep aids nor hormonal contraceptives or hormonal replacement therapy, which may affect the generalizability of our results.

In conclusion, the results of this study add to the understanding of melatonin levels in middle‐aged women living in socioeconomically distressed communities, both with stable HIV infection and without HIV infection. Assessing endogenous melatonin levels in such women is important given melatonin's role in sleep and circadian regulation and its anti‐inflammatory properties (Cho et al., 2021; Reiter et al., 2017). These data lay the foundation for exploration of the longitudinal consequences of endogenous melatonin levels on inflammatory‐based diseases (Forman et al., 2010; McMullan et al., 2013; Schernhammer & Hankinson, 2009) including those with higher prevalence among women aging with HIV and may provide insight into potential therapies.

## CONFLICT OF INTEREST STATEMENT

Dr. Burgess serves on the scientific advisory board for Natrol, LLC and is a consultant for F. Hoffmann‐La Roche Ltd. All other authors declare no conflicts of interest.

### PEER REVIEW

The peer review history for this article is available at https://publons.com/publon/10.1002/brb3.3206.

## Data Availability

The data that support the findings of this study are available on request from the corresponding author. The data are not publicly available due to privacy or ethical restrictions.
